# Supplementary material for: Real World Use of Tixagevimab/Cilgavimab Pre-Exposure Prophylaxis of COVID-19 in Immunocompromised Individuals: Data from the OCTOPUS Study
Source: Vaccines (Basel). 2024 Jul 17;12(7):784. doi: 10.3390/vaccines12070784 (PMC11281604; doi:10.3390/vaccines12070784)

Supplementary Material

Supplemental Figure S1. Flowchart on patients' disposition and study's timepoint.

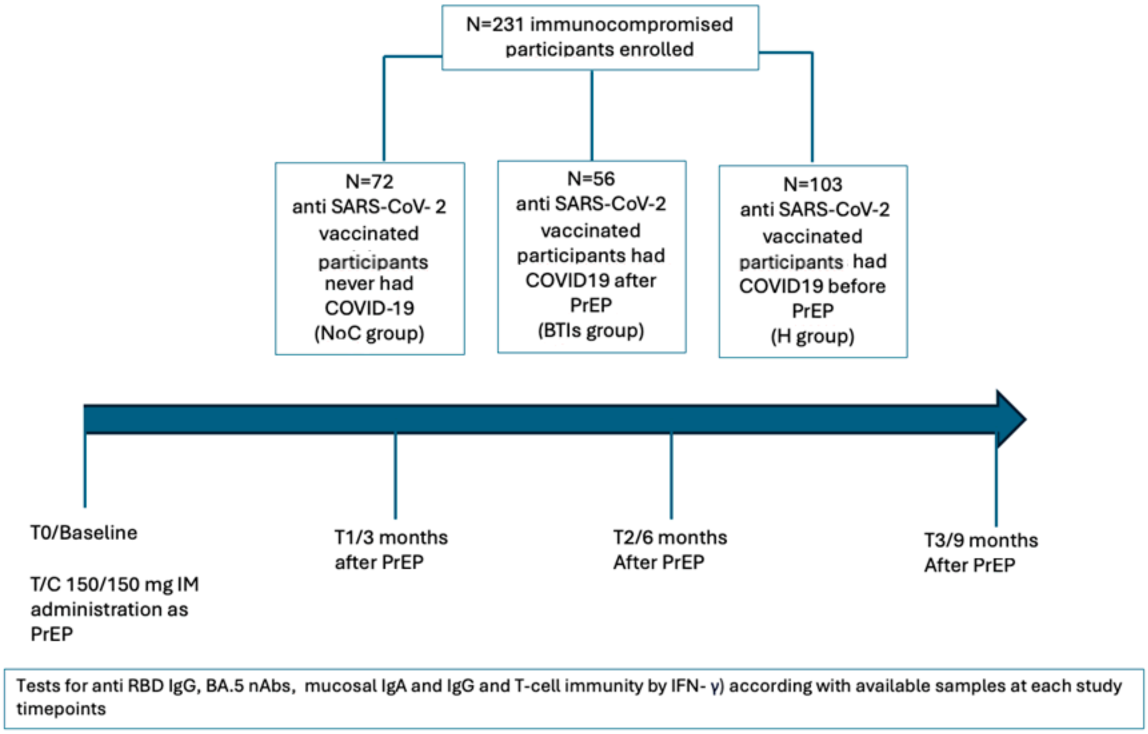

Abbreviations: NoC, No COVID-19; BTIs, Breackthrough Infections; H, Hybrid immunity; PrEP, anti COVID-19 pre-exposure prophylaxis; T/C, tixagevimab/cilgavimab; IM, intramuscularly; anti-Receptor Binding Domain (RBD); nAbs, neutralizing antibodies; IFN- γ, interferon- γ)

Supplemental Figure S2. Anti-SARS-CoV2 IgA levels measured in saliva in BTIs. Dot lines represent the cut-off of the assays. Kruskal Wallis and Dunn's tests were performed for statistical comparison. Red lines represent geometric mean values.

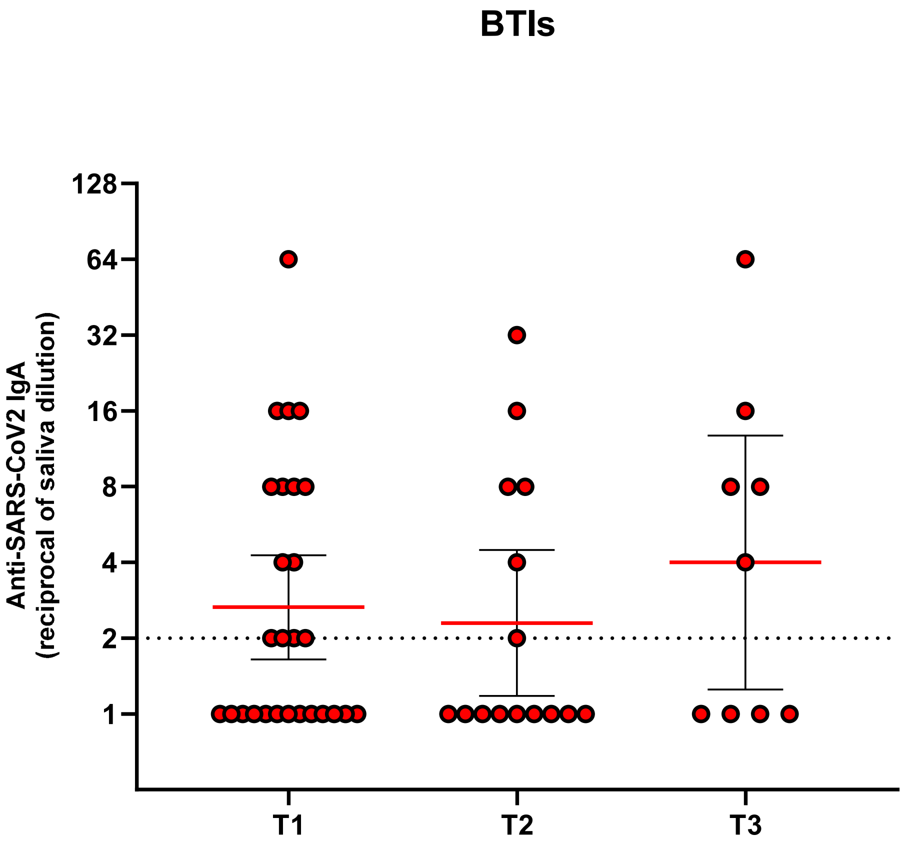

Supplement: Supplementary file 1 [file vaccines-12-00784-s001.zip › vaccines-3061601-supplementary.pdf]
